# Optimal Decision Model for Sustainable Hospital Building Renovation—A Case Study of a Vacant School Building Converting into a Community Public Hospital

**DOI:** 10.3390/ijerph13070630

**Published:** 2016-06-24

**Authors:** Yi-Kai Juan, Yu-Ching Cheng, Yeng-Horng Perng, Daniel Castro-Lacouture

**Affiliations:** 1 Department of Architecture, National Taiwan University of Science and Technology (NTUST), Taipei 106, Taiwan; rik@mail.ntust.edu.tw (Y.-K.J.); perng@mail.ntust.edu.tw (Y.-H.P.); 2 School of Building Construction, Georgia Institute of Technology, Atlanta, GA 30332, USA; daniel.castro@coa.gatech.edu

**Keywords:** decision support system, hospital, sustainable renovation, software tools, adaptive reuse

## Abstract

Much attention has been paid to hospitals environments since modern pandemics have emerged. The building sector is considered to be the largest world energy consumer, so many global organizations are attempting to create a sustainable environment in building construction by reducing energy consumption. Therefore, maintaining high standards of hygiene while reducing energy consumption has become a major task for hospitals. This study develops a decision model based on genetic algorithms and A* graph search algorithms to evaluate existing hospital environmental conditions and to recommend an optimal scheme of sustainable renovation strategies, considering trade-offs among minimal renovation cost, maximum quality improvement, and low environmental impact. Reusing vacant buildings is a global and sustainable trend. In Taiwan, for example, more and more school space will be unoccupied due to a rapidly declining birth rate. Integrating medical care with local community elder-care efforts becomes important because of the aging population. This research introduces a model that converts a simulated vacant school building into a community public hospital renovation project in order to validate the solutions made by hospital managers and suggested by the system. The result reveals that the system performs well and its solutions are more successful than the actions undertaken by decision-makers. This system can improve traditional hospital building condition assessment while making it more effective and efficient.

## 1. Introduction

Middle East respiratory syndrome (MERS), severe acute respiratory syndrome (SARS) and avian influenza are three newly emerged serious infections with pandemic potential [1,2]. These pandemic infections and diseases usually cause considerable loss of lives, resulting in increased social trepidation [1,3,4]. The role of a hospital is basically to be a line of defense against the spread of these infections and diseases. However, hospitals with inadequate building facilities have unexpectedly become the most hazardous areas, zones of defenselessness that undermines the entire medical and healthcare system.

Poor initial facility design and construction with little maintenance often result in a deterioration of the health infrastructure, a situation that may pose a danger to the health and safety of both patients and staff [5]. MERS and SARS, for example, are both serious respiratory diseases, and their outbreaks are closely related to poor indoor air quality that has resulted from inadequate ventilation in hospitals [6,7,8]. Indoor climate control system issues, such as improvements in noise control, air quality, ventilation, and lighting conditions, are receiving increased attention. Therefore, hospital managers should seriously consider the possibility of renovating existing hospital facilities, installing an improved HVAC system, and other indoor climate control systems, to decrease the spread of infections and to upgrade the medical environment through sustainable building renovation.

Building energy consumption in hospitals is another important issue related to sustainability. The building industry is one of the largest contributors to energy and material use worldwide [9]. In the commercial sector, energy use in hospitals accounts for 9% of the total consumption in USA, 11% in Spain, and 6% in UK, respectively. However, in terms of energy use intensity, hospital buildings are second only to restaurant buildings, and HVAC is the main end use with approximately half of the energy use in buildings [10]. Improving the energy efficiency of existing hospital buildings while maintaining high standards for the medical environments has been regarded as a priority for building renovation [11,12].

Various assessment tools are available to assist designers, developers and regulatory bodies in reducing the negative impacts of contemporary multi-housing subdivision projects in industrialized countries [13]. Many studies have developed a number of methods and models for evaluating existing building conditions and supporting decisions pertaining to building renovation, such as TOBUS for offices [14,15], XENIOS for hotels [16], IEA Annex 36 for educational buildings [17], EPIQR and other decision making tools for residential buildings [18,19,20]. Some studies have proposed strategies to deal with waste pollution and diseases spread in built environments [21,22,23,24]. However, there are three notions particularly noteworthy from this previous research. First, most of these methods and models have not been tested for hospital building types; compared to other building types, the integration of facilities and equipment in hospitals is more complicated. Second, these methods and models have paid most attention to understanding or predicting energy usage for buildings. The discussion of applicable renovation strategies for improving building performance is relatively limited [14]. Third, there are many studies related to infection surveillance systems for healthcare in various countries [25,26,27], but many highly infectious diseases (HIDs) spread rapidly when infection control measures are poorly implemented [28]. Improvement and maintenance of healthcare facilities are crucial to minimize the problem of a major breakdown and to satisfy the end user [29].

The objective of this research is to develop an integrated decision support system to assess the current building environmental conditions in hospitals, to provide sustainable hospital renovation and maintenance strategies for decision makers, and to improve the quality of the hospital environment to prevent nosocomial infections by renovation. The system, considering the trade-offs between renovation benefits and costs, is developed based on a hybrid approach combining A* and genetic algorithms (GA). The risks of nosocomial infections can be effectively decreased and the energy consumption in hospitals can be substantially reduced with the implementation of renovation strategies, which may be helpful for the future development of hospitals and medical environments.

## 2. Overview of Hospital Renovation and Energy Use

The lifespans of components in a building vary dramatically. Structure and exterior systems, for instance, may last for more than 50 years while infill systems, such as interior installation, electrical or mechanical systems, may sustain for only 3–20 years. There can be several renovations of infill systems while the structure system is still in fine condition [30]. Adequate maintenance should be provided throughout the entire lifetime of a building to ensure that the building and its facilities meet the requirements stated by the users and specifications [31]. Periodic renovation can also provide opportunities to upgrade the internal and external environments, increase the value of existing buildings, provide more modern accommodations, and attract new owners or occupants [32]. Hospitals, compared to other building types, involve facilities and equipment that are relatively complicated. Furthermore, the emphasis on the integration of space operation and system functionality has increased the difficulty in managing these facilities and equipment. A systematic approach to managing these periodic maintenance and renovation activities is imperative. 

Taking sustainability into account, hospital facilities and equipment consume appreciable energy to maintain their health care functions. The yearly average energy use intensity (EUI) for hospital buildings is estimated at 389.8 kBtu/ft^2^, second only to supermarket/grocery buildings estimated at 480 kBtu/ft^2^ in the United States [33]. Many studies have stressed that efficient design for facilities and appropriate adoption of equipment can save energy during an operation life-cycle [34,35,36]. Therefore, through sustainable renovation, hospital managers can implement cost-effective measures, transforming their buildings so that they become more resource-efficient and environmentally sound for the long run.

## 3. Criteria for Sustainable Hospital Building Renovation Assessment and Rating Rules

### 3.1. Criteria for Sustainable Hospital Building Renovation

Leadership in Energy and Environmental Design (LEED) is one of the most prestigious building environmental schemes in the world [37]. LEED for Healthcare, developed by the U.S. Green Building Council (USGBC), represents close collaboration between the Green Guide for Health Care (GGHC) and USGBC, includes seven major credits: sustainable sites, water efficiency, energy and atmosphere, materials and resources, indoor environmental quality, innovation in design, and regional priority. LEED for Healthcare addresses design and construction activities for both new buildings and major renovations of existing buildings. These guidelines provide healthcare owners to build green and maximize both economic and environmental performance for healthcare facilities [38].

The Green Guide for Health Care (GGHC) is a voluntary, self-certifying metric toolkit of health-based best practices, customized for the healthcare sector. Designers, owners, and operators can use the GGHC to guide and evaluate progress toward high performance healing environments. Healthcare facilities present unique opportunities for developing and implementing sustainable design, construction, and operations practices. Issues such as 24/7 operations, energy and water use intensity, chemical use, infection control, and regulatory requirements can pose significant obstacles to implementing sustainability protocols. The Green Guide directly incorporates the language of a parallel LEED credit, referencing credits in the LEED systems for New Construction, Existing Buildings—Operations and Maintenance and Commercial Interiors. In some cases, existing LEED credits have been modified to respond to the unique needs and concerns of healthcare facilities. The Green Guide has already supported 311 registered hospital projects [39].

The Building Research Establishment Environmental Assessment Method (BREEAM) is an environmental assessment method for new or existing buildings in the UK and also is one of the most commonly used building performance rating systems worldwide [37]. BREEAM Healthcare is widely used in the environmental assessment of the sustainable performance of healthcare buildings, such as teaching hospitals, specialist acute hospitals, general acute hospitals, community hospitals, cottage hospitals, mental health hospitals/unit, learning disability units, GP (General practitioner) surgeries and health centers and clinics. Criteria include management, health and wellbeing, energy, transport, water, materials, waste, land use and ecology, pollution, and innovation [40].

LEED for Healthcare, GGHC, and BREEAM (Healthcare) differ in terminologies, structures, performance assessments, relative importance of environmental performance categories and documentation requirements for certification, [37,41,42]. However, any of their standards can provide a good reference to establish criteria to evaluate the renovation sustainability of hospital buildings. “Indoor environmental quality” credits of LEED for Healthcare and “Health and wellbeing” credits of BREEAM (Healthcare), for example, are exclusive criteria for hospital buildings. These criteria are highly related to assessment of infection control, patients’ contaminants management, and indoor air quality control in the hospital [38,40]. In this study, sustainable renovation means upgrading or improving building facilities and equipment with regards to environmental sustainability and ensuring that the renovation strategies are cost-effective. The physical condition and space utilization condition assessments of building systems that are not directly related to environmental sustainability are excluded from this study when considering these sustainable renovation strategies. Table 1 shows a comparison of sustainable hospital building renovation criteria and the credits from LEED, BREEAM and GGHC. The focus of this study is to evaluate environmental conditions of existing buildings and to recommend an optimal scheme of sustainable renovation strategies. Therefore, criteria, such as, location, transportation, regional priority and innovation, are not considered in the research. Table 2, based on the Table 1, shows integrated criteria, sub-criteria, and assessment items for sustainable hospital building renovation. Among the proposed criteria, the category of “health and well-being” is added to highlight its importance to healthcare and medical environments.

### 3.2. Assessment and Rating Rules

In order to quantify the quality, six experienced building renovation contractors and experts were interviewed to assess these criteria and transfer them into scores. The cost of each renovation strategy was also determined by these contractors and experts. Table 3 shows the principles of rating rules and the assessment items including cost and score information of the second criterion: water efficiency. Thirty current condition questions (assessment items) with basic building information are provided for the users to assess the current hospital building condition. The principles of rating rules are separated into two parts: current condition assessment scores and improvement scores. In order to quantify the quality of renovation strategies, improvement scores are also simply rated by three levels according to the renovation involvement. “Low level” means that taking these actions can lead to the full restoration to original designed functioning levels (the score of 1). For example, “tap with water-efficiency” had a score of 1 because it can reach the basic requirement of water conservation. “Medium level” means taking these actions can not only restore full function of facilities or equipment but also increase their service life or durable capacity (the score of 1.5) (e.g., the renovation can improve its functional performance superior to original designed function). For example, “tap with automatic sensor” had a score of 1.5 because it can achieve more water saving amount than “tap with water-efficiency”. “High level” means taking these actions can lead to the most sustainable benefits, such as energy saving, water conservation, and other benefits related to environmental sustainability, for the environment (the score of 2). For example, “tap with water-efficiency and automatic sensor” had a score of 2 because it combines two functions of tap water saving techniques, which can conserve much more water.

**Figure 1:**
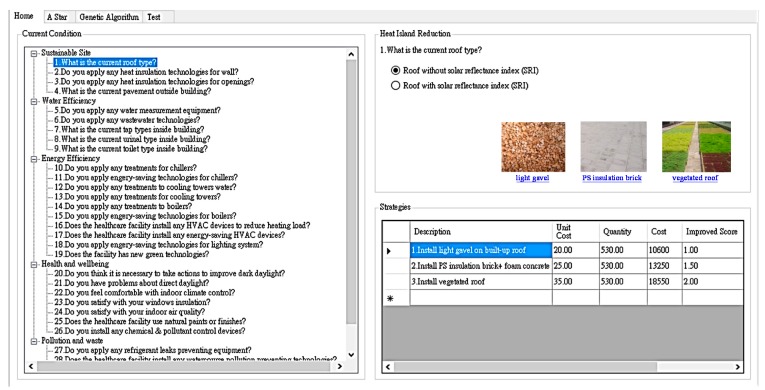
Interface of sustainable condition assessment.

Figure 1 shows the interface of condition assessment system providing assessment items and their corresponding improvement actions with photos and explanations. Flexible data modules also record cost and improved score information for each renovation strategy. The final scores are the sum of the assessment score for each criterion and the improved score for each renovation strategy, presenting the sustainable renovation level of the hospital building.

## 4. Methods

**Figure 2:**
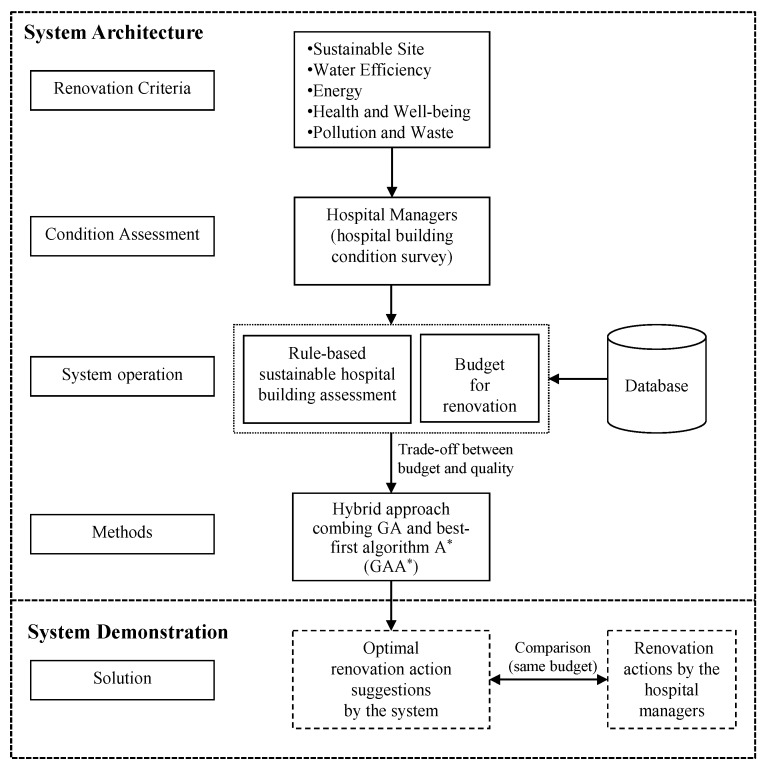
Architecture of decision support system.

GA combining A* is used as method of the decision support system in this research. The framework of the decision support system involves four major processes as shown in Figure 2: (1) Condition assessment, assess the sustainability level of the hospital building based on the criteria; (2) System operation, calculate the varied refurbishment solutions; (3) Methods, provide renovation actions by adopting a hybrid approach algorithm that analyses the trade-off between the user’s preferred budget and expected improvement level; and (4) Solution, compare the renovation solution actions of a real project and decisions suggested by the system.

### 4.1. Genetic Algorithm and A* Search Algorithm

GA as an efficient analytic tool for solution of optimization problems is shown to be effective at solving large and complicated problems in an adaptive way guided by the equivalent biological evolution mechanisms of reproduction, crossover and mutation [43]. By giving more chances to the better elements to have offspring in the next generation, the GA facilitates an evolutionary process in which elements in a population progressively improve over time [44]. Genetic algorithms (GA) now are well known by their great operations in combinatorial optimization [45]. However, one of the major drawbacks in a traditional GA is a random process, which generates the initial population for each chromosome [46], and each chromosome of traditional GA in the initial population is generated randomly. Previous experimental results have shown that the GA does not always result in good solutions due to the random method. The reason is that most of the randomly generated chromosomes have a poor total flowtime and thus pass unfavorable traits to their offspring [47].

Of all search strategies used in problem solving, one of the most popular methods of exploiting heuristic information to cut down search time is the informed best-first strategy [48]. A* search is the most widely known form of best-first search and optimally efficient for any given consistent heuristic. The concept of A* is to explore a graph through traversing to find the optimal path according to its specific rules. A* is able to predict how close the end of a path is to a solution with its heuristic function, so that paths which are judged as being closer to a solution are extended first [49]. However, A* is not practical for many large-scale problems. A* usually runs out of space long before it runs out of time because it keeps all generated nodes in memory [49], and not having a mutation mechanism like GA, does not guarantee that the optimal solution can always be found effectively [14].

### 4.2. Hybrid Algorithm (GAA*) for Renovation Solution Optimization

The task of this system is to automatically search for the most cost-effective (lower cost and higher quality) renovation strategies that belong to each assessment item. However, all the combinations of renovation strategies exceed more than three hundred trillion, which is an astronomical figure infeasible for manual search optimization. Adopting an effective and robust algorithm is therefore critical for success of the system. Some study has demonstrated the potential of using the hybrid algorithm of GAA* (Genetic algorithm and A* search algorithm) to deal with office building renovation projects. GAA* can be a promising approach for solving the large-scale zero-one programming determinate problem effectively [14]. In the present work, the optimization problem can be formulated as follows:

(1) $$Max\;Z_{1}=\sum_{i=1}^{m}\sum_{j=1}^{n}X_{ij}\cdot S_{ij}$$

(2) $$Min\;Z_{2}=\sum_{i=1}^{m}\sum_{j=1}^{n}X_{ij}\cdot C_{ij}$$

subject to:

(3) $$\sum_{i=1}^{m}\sum_{j=1}^{n}X_{ij}\cdot C_{ij}\leq \;C_{T}$$

(4) $$\sum_{j=1}^{n}X_{ij}=1\text{ For }i=\;1,\;2,\;\ldots ,n$$

where *Z*_1_ and *Z*_2_ are the objective functions maximizing the total score and minimizing the total cost of selected actions, respectively; *Sij* and *Cij* represent the score and cost for the ith action of the jth assessment item, respectively; and *Xij* is a variable (*Xij* = 1 means that the ith action is selected under the jth assessment item). The above functions are constrained under the renovation budget (*C_T_*) of the decision-maker.

**Figure 3:**
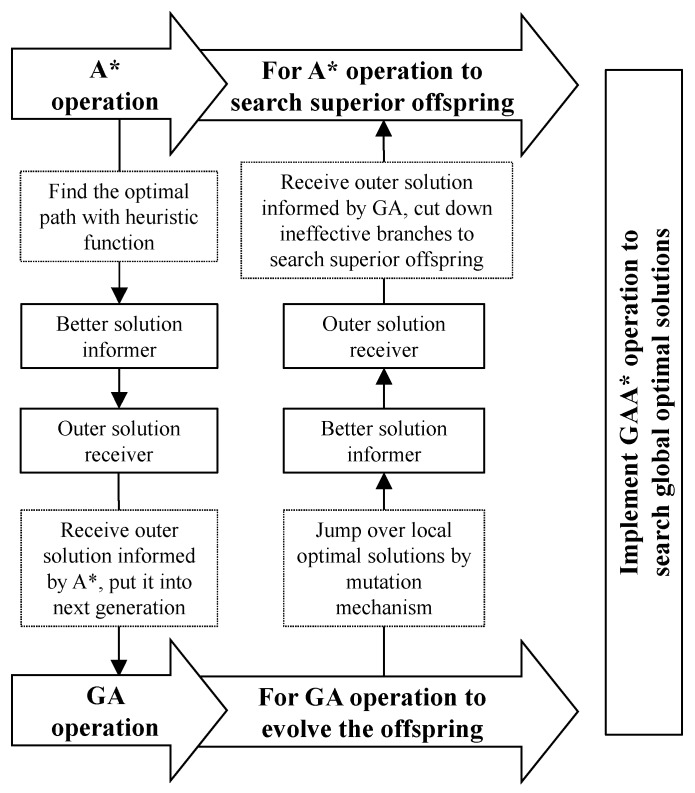
Operation concept of Genetic algorithm and A* (GAA*).

The system is developed under the Java Server Pages (JSP), Java environment, Apache Tomcat web container, and MySQL database. Figure 3 depicts the complementation of A* and GA of this hybrid approach. A* and GA are two individual operation approaches but as A* searches for a better solution, GA could adopt this solution to offspring evolution. Hence, using A* could overcome ineffective initial population selection, which is the major defect of GA. GA could also offer better solutions to support A* to jump over local optimal solutions by its mutation mechanism, which has greatly improved the robustness of the algorithm. This complementation continues to repeat until the results reach the optimal solution.

## 5. Results

### 5.1. A Briefing on a Vacant School Building Converting to a Public Community Hospital

Building reuse was the primary strategy and trend for urban development in developed countries during the 1960s and 1970s. Rather than contributing to waste through the demolition of old buildings and the construction of new buildings, reusing buildings extends a building’s life and facilitates environmental sustainability while providing social and economic benefits to the society [50,51]. In developed countries, 95%–97% of buildings are existing buildings. Reusing building structures, such as through refurbishments, infrastructure additions, or reconstruction, requires merely 50%–75% of the time and 50%–80% of the costs entailed in constructing new buildings [52]. Therefore, adaptive reuse of vacant buildings is an efficient way to use obsolete existing buildings by “recycling” them in-situ, thereby providing them a new functional purpose [53].

Combined with declining birth rates, societies around the world are now facing the challenges of declining birth rates combined with rapidly aging population [54]. In Taiwan, the birth rate in 2010 was the world’s lowest [55]. As a result of this declining, the number of students there has also been decreasing each year. In fact, it is estimated that 30% of middle school spaces will be unoccupied by the end of 2028 [56]. The aging population in Asia is increasing, from 10% in 2009 to 30% in 2050. While healthcare service is essential for the well-being of elderly people in terms of personal care, nursing, life threatening illness, health consulting, and contingency help [57], transportation convenience should also be considered for elders [54,58]. As in societies and their governments worldwide, Taiwan should also prepare to support the growth of these trends [59]. Instead of large and teaching hospitals which are provided with interns, more sickbeds and long-term medical care, community hospitals provide short-term general specialist and hospitalization, including referral resource, first aid and emergency, hospitalization service, prevention and public education, and basic medical and public health service for surrounding communities. The needs of the society must be addressed for vacant public buildings conversion [60]. Complying with the tendency of declining birth rates and rapidly aging populations, the Taiwan government has actively proposed building reuse policies with respect to converting vacant public school buildings to other public facilities, such as community hospitals, in these years [61]. Middle schools in Taiwan, for example, are usually located within communities, and reusing these school buildings as they become vacant by converting them to public community hospitals is more convenient for elders and children who need more frequent general health examinations and medical treatments. Some classrooms in schools can be easily converted to medical consultation rooms and the others can be used for public medical education and service without demolition. Relevant projects are going to be conducted one after another.

**Figure 4:**
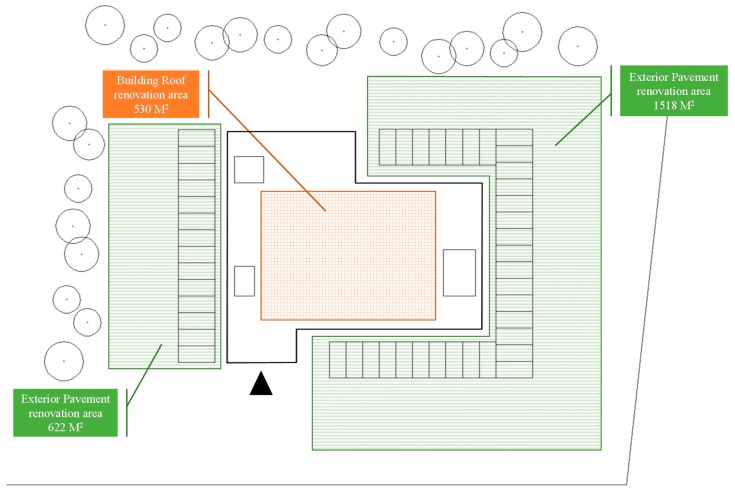
Site plan of the simulated school building project.

The simulated vacant school building renovation project is located in the south area of Taipei city, the site plan and outdoor renovation area of this case as shown in Figure 4. This reused school building was built to last more than 20 years and was unused for a few years because of the declining birth rate. In terms of sustainability, this building has many existing problems including improper insulation in roofs, unsuitable exterior walls, inadequate indoor ventilation systems, inefficient water appliances, and insufficient greenery. Rehabilitation of the environment and facilities is required for the renovation project. The renovation budget on environmental issues for this building with a total floor area of 5210 m^2^ with four stories and one basement is US$700,000. Table 4 describes the current condition of the school building. A score of 1 means the current condition is satisfied; a score of 0 means the current condition requires renovation. The total assessment score of the current condition is 8.

### 5.2. Comparison Result between the Simulated Project and the System

**Figure 5:**
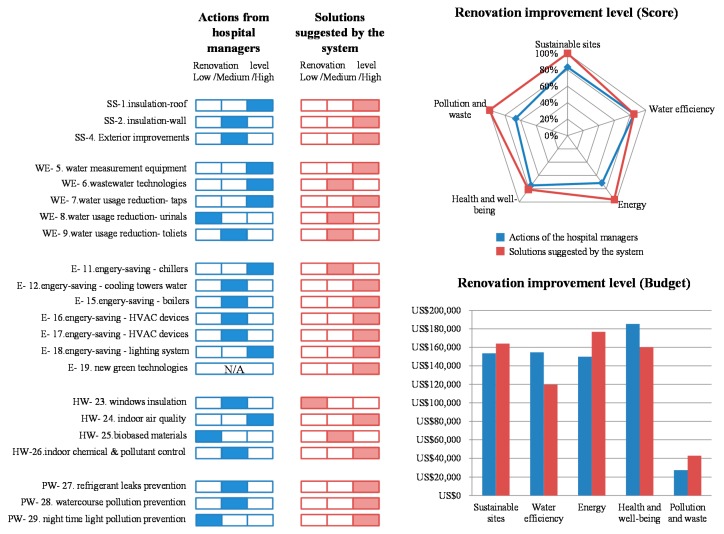
Comparison result between the project decided by hospital managers and the system.

Table 5 illustrates the differences in renovation actions for the simulated project between building managers and the solutions proposed by the system. Figure 5 presents the comparison between actions made by hospital building managers and solutions suggested by the system. There are some differences in adopting renovation strategies for these two compared items. In the criterion of sustainable sites, the solutions suggested by the system cost more money than what hospital managers suggested because of high level actions for wall insulation technologies and eco-pavement. In the criterion of water efficiency, the hospital managers take a high level strategy for wastewater technologies that charges a costly budget, thereby increasing the total cost amount. In the criterion of energy, the system is adopting new green technology—building energy management system which is classified to high level, while the hospital managers take no action to improve the energy performance. In the criterion of health and well-being, hospital managers are trying to choose actions to improve the occupant environment that also raise the budget. For example, hospital managers choose the window insulation strategy with a high cost. In the criterion of pollution and waste, the system pays attention to prevention of refrigerant leak, watercourse pollution, and night time light pollution while hospital managers only take into account of the first two sub-criteria. Therefore, the total score yielded by the system (40.5) is higher than that of the project decided by hospital managers (33.5), as shown in Table 5.

As for the actions made by hospital building managers, with the budget of US$ 671,166, a series of renovation strategies were adopted, yielding an improvement in the score from 8 to 33.5. This system, using the same budget as the actual renovation, achieved a score of 40.5. The actual cost (US$ 663,686) for implementing these renovation strategies suggested by the system is even lower than the original budget. In addition, by using GAA* for the optimal searching process, these solutions can be found in 0.05 s (the limit of generation is set at 500; the original population size is set at 1000; crossover and mutation rates of GAA* operation are 0.66 and 0.25, respectively), and the improvement score increases from 8 to 40.5. Comparing the result of actions made by hospital building managers and solutions suggested by the system, the system can provide more cost-benefit solutions (lower cost and higher improved score), which can prove the robustness and efficiency of the system, as well as satisfy client expectations.

## 6. Conclusions and Suggestions

Hospitals are the effective means for preventing the spread of newly emerged infections and diseases. Without well-established building facilities and healthcare systems, hospitals can become hazardous workplaces with dangers to the health and safety of both patients and staff. A systematic and innovative approach that formulates a procedure to upgrade existing facilities and systems as well as improve energy performance through sustainable renovation is a win-win strategy for hospital planning and management in the future.

Rehabilitating unused buildings by adjusting the service values to extend building life cycles is an efficient way to achieve a sustainable construction development globally. Much attention to the assessment of reusing vacant buildings is still required. Researchers should be concerned not only with energy consumption reduction but also with adopting more energy-efficient renovation strategies when reusing vacant buildings to minimize environmental impacts. The feasibility of a conversion project also should be considered for the future operation. Balanced scorecard (BSC), as a management innovation integrating financial and non-financial performance measures in light of organizational strategy, has been widely adopted by various organizations [62]. Many building delivery organizations are now using the balanced scorecard to evaluate their performance [63]. In the future, balanced scorecard can be used for acceptability evaluation of this conversation project, which is not included in this research.

This study presents an integrated decision support system to assess the current building conditions and to provide sustainable renovation strategies for hospital decision makers. As for the system architecture, an assessment mechanism is first used to diagnose the current condition of the building. Second, an optimization mechanism adopting GAA* is developed to optimize renovation strategies within several constraints. Finally, a simulated vacant school building converting to a public community hospital renovation project is introduced to validate the proposed system. The solutions suggested by the system are shown to be superior to the results from the project decided by the hospital managers, not only in budget utilization, but also of quality improvement. In this study, reusing and renovating vacant buildings can not only be helpful to reduce energy consumption within the buildings, but also be friendly to the environment and beneficial to nearby communities.

Although the proposed system demonstrated its robustness and efficiency for decision-making, the system still had certain limitations. First, the system is developed for hospital managers, and most of them are non-experts in architecture/engineering/construction fields; providing a simplified method for current condition assessment and restoration actions is necessary. This simplified process, however, might cause the system to be incomprehensive. Second, the system was only tested by hospital buildings; although the data modules and interfaces of the system provide managers with great flexibility to clarify, modify, and customize the assessment criteria and renovation strategies to apply to different building types, future efforts to examine its feasibility on different building projects are highly suggested. Third, the system paid more attention to environmental sustainability issues, renovation actions resulted from functionally spatial adjustment were not considered. Finally, only general specification of materials, resources and equipment for hospital buildings were included in the system; future research could continuously expand practical or specific data to establish a more integrated decision support system. 

## Figures and Tables

**Table 1:** Sustainable hospital building renovation criteria with credits of LEED, BREEAM and GGHC cross index.

Sustainable Hospital Building Renovation Criteria	LEED for Healthcare	BREEAM (Healthcare)	GGHC
	Regional priority	Management	Transportation operations
		Transport	
Sustainable sites	Sustainable sites	Land use and ecology	Environmental service
Water efficiency	Water efficiency	Water	Water efficiency
Energy	Energy and atmosphere	Energy	Energy and atmosphere
Health and well-being	Indoor environmental quality	Materials	Materials and resources
	Materials and resources	Health and wellbeing	Chemical management
Pollution and waste		Waste	Waste management
		Pollution	
	Innovation	Innovation	Innovation

**Table 2:** Sustainable hospital building renovation criteria.

Criteria	Sub-Criteria	Assessment Items
Sustainable sites	Heat island reduction	Building roof, wall and opening
	Exterior improvement	Paving type
Water efficiency	Water performance measurement	Water measurement equipment
	Wastewater technologies	Water technologies
	Water use reduction	Current urinal and toilet type
Energy	HVAC system	Energy-saving technologies and treatments for chillers, cooling towers, boilers and HVAC devices. Energy- reducing heating load technologies for HVAC devices.
	Lighting system	Lighting systems technologies
	Innovative technologies	New green technologies
Health and well-being	Daylight	Daylight control
	Occupant comfort	Climate control, windows insulation, indoor air quality
	Indoor chemical and pollution control	Chemical and pollution control devices
Pollution and waste	Pollution	Preventing technologies or devices for refrigerant, water course and night time light pollution.
	Waste	Waste storage management

**Table 3:** Example of assessment items under the Energy criterion with principles of rating rules.

Criteria	Current Condition	Assessment Description		Assessment Score (before Renovation)	Improved Score (after Renovation)
Water Efficiency—Water use reduction	7. What are the current tap types inside facility?	Yes (satisfy current conditions)		1	0
		No (need to take renovation actions)		0	1–2
		Renovation strategies	Renovation level	Cost (per unit)	Improved score
		1. Tap with water-efficiency	Low level solutions for renovation	100 per each	1
		2. Tap with automatic sensor	Medium level solutions for renovation	140 per each	1.5
		3. Install “1” + “2”	High level solutions for renovation	200 per each	2

**Table 4:** Assessment of current condition of the hospital building.

Criteria	Current Condition	Assessment Scores
Sustainable site		
Heat Island Reduction—roof	1. Current building roof materials is not with solar reflectance index (SRI)	0
Heat Island Reduction—wall	2. No heat insulation technologies for walls	0
Heat Island Reduction—opening	3. Heat insulation technologies for openings are totally adopted	1
Exterior improvements	4. Current pavement outside building is not eco- pavement	0
Water efficiency		
Water performance measurement	5. Water measurement equipment is not adopted	0
Wastewater technologies	6. No wastewater technologies	0
Water use reduction	7. Efficient tap type is not totally adopted	0
	8. Efficient with less water urinal type is not totally adopted	0
	9. Efficient toilet type is not totally adopted	0
Energy		
HVAC system	10. Treatment for chillers is adopted	1
	11. No energy-saving technologies for chillers	0
	12. Cooling towers water treatment is not adopted	0
	13. Treatment for cooling towers is adopted	1
	14. Treatment for boilers is adopted	1
	15. No energy-saving technologies for boilers	0
	16. HVAC devices used to reduce heating load are not installed	0
	17. No energy-saving technologies for HVAC devices	0
Lighting system	18. Energy-saving technologies for lighting system is not adopted	0
Innovative technology	19. No new green technologies in the building	0
Health and wellbeing		
Daylight	20. Not necessary to improve daylight piping to interior	1
	21. No direct daylight	1
Occupant comfort	22. Indoor climate control is fine	1
	23. Window insulation is required	0
	24. Improving IAQ (indoor air quality) is required	0
Biobased materials	25. No natural paints or finishes	0
Indoor chemical & pollutant control	26. Indoor chemical & pollutant control devices are not totally installed	0
Pollution and waste		
Pollution	27. No refrigerant leak preventing equipment	0
	28. No watercourse pollution preventing technologies	0
	29. Night time light pollution preventing technologies are not totally adopted	0
Waste	30. Good management for recyclable waste storage is adopted	1
Total assessment score		8

**Table 5:** Comparison of renovation action solutions.

Criteria	Improved Items	Actions from Hospital Building Managers (M)	Actions from the System (S)	Improved Scores (M/S)
Sustainable site				
Heat Island Reduction—roof	1. Building roof type	Install vegetated roof	Install vegetated roof	2/2
Heat Island Reduction—wall	2. Wall insulation technologies	Install heat insulation coating	Install heat insulation board	1.5/2
Exterior improvements	4. Eco-pavement	Install Porous unit paving	Install Turf blocks	1.5/2
Water efficiency				
Water performance measurement	5. Water measurement equipment	Install water meters + automatic leak detectors	Install water meters + automatic leak detectors	2/2
Wastewater technologies	6. Wastewater technologies	Install rainwater capture system + graywater recycling system + wastewater treatment system	Install rainwater capture system + wastewater treatment system	2/1.5
Water use reduction	7. Efficient tap type	Tap with water-efficiency and automatic sensor	Tap with water-efficiency and automatic sensor	2/2
	8. Efficient with less water urinal type	Install waterless urinal	Install water-free urinal	1/1.5
	9. Efficient toilet type	Install vacuum toilet system	Install vacuum toilet system	1.5/1.5
Energy				
HVAC system	11. Energy-saving technologies for chillers	Install Variable Water Volume System + Variable Air Volume System + Chiller amount controlling + use of ice tanks + Heat recovery chiller system	Install Variable Water Volume System + Variable Air Volume System + Chiller amount controlling + use of ice tanks	2/1.5
	12. Cooling towers water treatment	Medicament treatment	Ozone treatment	1.5/2
	15. Energy-saving technologies for boilers	Install CO_2_ sensors + Warm-keeping of pipes + air preheater + Cool-condensed water recycling	Install CO_2_ sensors + Warm-keeping of pipes + air preheater + Cool-condensed water recycling + waste heat recovery	1.5/2
	16. Energy-reducing heating load technologies for HVAC devices	Install CO_2_ sensors + Outside Air Economizer	Install CO_2_ sensors + Outside Air Economizer + Heat recovery chiller system	1.5/2
	17. Energy-saving technologies for HVAC devices	Ductwork insulation and sealing + Variable Water Volume System + Variable Air Volume System + Variable Refrigerant Volume System + Cooling water recycling	Ductwork insulation and sealing + Variable Water Volume System + Variable Air Volume System + Variable Refrigerant Volume System + Cooling water recycling + Thermal storage system	1.5/2
Lighting system	18. Energy-saving technologies for lighting system	High performance lamps and electronic ballasts + Illumination meters and illumination rationalization + Manual-on, automatic-off sensors	High performance lamps and electronic ballasts + Illumination meters and illumination rationalization + Manual-on, automatic-off sensors	2/2
Innovative technology	19. New green technologies	N/A	Building energy management system	0/2
Health and wellbeing				
Occupant comfort	23. Windows’ insulation	Install double-glazed with low-e coatings	Install solar-control window film	1.5/1
	24. Improving IAQ (indoor air quality)	Temperature auto-regulator + Air filter maintenance	Temperature auto-regulator + Air filter maintenance	2/2
Biobased materials	25. Natural paints or finishes	Natural paints and adhesive	Apply low-odor finishes	1/1.5
Indoor chemical & pollutant control	26. Indoor chemical & pollutant control devices	Utilize entryway systems + Isolated exhaust system for pollutant areas	Install and maintain air filtration media + Utilize entryway systems + Isolated exhaust system for pollutant areas	1.5/2
Pollution and waste				
Pollution	27. Refrigerant leak preventing equipment	Install refrigerant leak detection meters	Ductwork sealing and install refrigerant leak detection meters	1.5/2
	28. Watercourse pollution preventing technologies	Install leaks detectors + Oil separators maintenance	Install leaks detectors + Oil separators maintenance + Shut-off valves fitted to the site drainage system	1.5/2
	29. Night time light pollution preventing technologies	Automatic-off timers for external lighting 23:00–07:00 (except for safety and security lighting)	Automatic-off timers for external lighting 23:00–07:00 (except for safety and security lighting) + Ultrared rays automatic-on for stairway and hallway at night	1/2
Total assessment score		33.5/44	40.5/44	33.5/40.5
Budget (US$)		671,166	663,686	
